# Intraoral Scanning as an Alternative to Evaluate the Accuracy of Dental Implant Placements in Partially Edentate Situations: A Prospective Clinical Case Series

**DOI:** 10.3390/jcm11195876

**Published:** 2022-10-05

**Authors:** Jan van Hooft, Guido Kielenstijn, Jeroen Liebregts, Frank Baan, Gert Meijer, Jan D’haese, Ewald Bronkhorst, Luc Verhamme

**Affiliations:** 1 Department of Dentistry, Radboudumc Nijmegen, Philips van Leydenlaan 25, 6525 EX Nijmegen, The Netherlands; 2 Department of Oral and Maxillofacial Surgery, Radboudumc Nijmegen, Geert Grooteplein Zuid 10, 6525 GA Nijmegen, The Netherlands; 3 3D Lab, Radboudumc Nijmegen, Geert Grooteplein Zuid 10, 6525 GA Nijmegen, The Netherlands

**Keywords:** oral implantology, intraoral scan, accuracy, cone-beam computed tomography, oral surgery

## Abstract

(1) Background: For years, Cone-Beam Computed Tomography’s (CBCT) have been the golden standard to evaluate implant placement accuracy. By validating Intraoral Scans (IOS) as an alternative to determine implant placement accuracy, a second CBCT could be avoided. (2) Methods: Using dynamic guided implant surgery, 23 implants were placed in 16 partially edentate patients. Preoperatively, both CBCT and IOS (Trios^®^ 3) were obtained and subsequently imported into DTX Studio™ planning software to determine the ideal implant location. A CBCT scan and an IOS including scan abutments were acquired immediately after placement. Both postoperative CBCT and postoperative IOS were used to compare the achieved implant position with the planned implant position and were projected and analyzed using the Implant Position Orthogonal Projection (IPOP) method. (3) Results: Mean differences between the CBCT and IOS methods on the mesio–distal plane were 0.09 mm (*p* = 0.419) at the tip, 0.01 mm (*p* = 0.910) at the shoulder, −0.55° (*p* = 0.273) in angulation, and 0.2 mm (*p* = 0.280) in implant depth. Mean differences between both methods on the bucco-lingual/bucco-palatal plane were 0.25 mm (*p* = 0.000) at the tip, 0.12 mm (*p* = 0.011) at the shoulder, −0.81° (*p* = 0.002) in angulation, and 0.17 mm (*p* = 0.372) in implant depth. A statistical analysis was performed using a paired t-test. All mesiodistal deviations between the two methods showed no significant differences (*p* > 0.05). Buccolingual/buccopalatal deviations showed no significant difference in implant depth deviation. However, significant differences were found at the tip, shoulder, and angulation (*p* < 0.05). These values are of minimal clinical significance. (4) Conclusions: This study supports the hypothesis that a postoperative IOS is a valid alternative for determining implant placement accuracy.

## 1. Introduction

When osseointegrated implants became introduced in dentistry, their primary role was to re-establish a loss of function. Later on, due to constant advancements in implant design, ameliorated implant surfaces, and the introduction of challenging treatment protocols, aesthetic demands became more relevant. Patients insisted on shorter treatment protocols with predictable results both from a functional and esthetical viewpoint. With these increasing demands and expectations, the role of preoperative implant planning also becomes more relevant.

Almost 25 years ago, computer-aided design and manufacturing was introduced in implant dentistry as a tool to enhance accuracy and precision to install dental implants. Currently, there are two pathways to implement this technology in clinical practice. One can either use a static approach using preprinted surgical templates or opt for dynamic guided surgery, which is also known as navigation surgery. The last one, which is the most recently developed, is based on motion-tracking technology. It enables the real-time visualization of both drills and fixture on the combined image of the preoperative Cone-Beam Computed Tomography (CBCT) and intraoral scan (IOS), where the planned location is also visible [1].

To evaluate the accuracy of implant installation, the gold standard is to make use of a postoperative CBCT [2,3]. Applying voxel-based matching [4,5], both pre- and postoperative CBCT are aligned on top of each other to measure the deviation between the actual position of the dental implants and their pre-surgical position in the planning software.

In recent years, the IOS was introduced in dental practices as a valid alternative for conventional impression protocols. To determine implant placement accuracy, a postoperative IOS could be a viable alternative to a postoperative CBCT. In this manner, the radiation load for patients is reduced by avoiding a postoperative CBCT and the associated radiation dose of 2 to 1000µSv (equivalent of 2 to 200 panoramic radiographs) [6].

Besides the IOS, there are other, non-invasive methods for determining implant placement accuracy. The photogrammetric method [7] can determine the implant’s location using photographs from multiple angles, and the implant is made of a cast from the patient’s jaw.

Another method is the contact scan method, where, by also using a postoperative cast of the patient’s jaw, the location of the placed implant is determined using a contact scanner.

However, both methods require extra steps, since, in a digital workflow, an IOS is necessary anyway to fabricate the dental prosthesis, and a (plaster) cast of the patient’s jaw is normally not necessary; moreover, these methods also require either dedicated cameras or a contact scanner, which are both not needed for regular treatment protocols.

The aim of this prospective clinical case series was to evaluate if a postoperative IOS is a reliable alternative to a postoperative CBCT to determine dental implant placement accuracy in an in vivo setting and to describe deviations between both methods.

## 2. Materials and Methods

### 2.1. Patient Selection

In total, 16 dentate patients, referred to the department of Oral and Maxillofacial Surgery at Radboudumc Nijmegen to install at least one dental implant, were enrolled in this study. Patients were excluded if they were suffering from active periodontal disease, severe bruxism, or when intravenous bisphosphonates were administered. All patients provided written informed consent. Patients were not selected regarding implant location. They were treated according to their specific desire to restore the edentulous area. The protocol was evaluated and approved by the ethical committee of Oost-Nederland (file nr 2020-6449) and performed according to the Declaration of Helsinki.

### 2.2. Preoperative Data Acquisition

**Figure 1:**
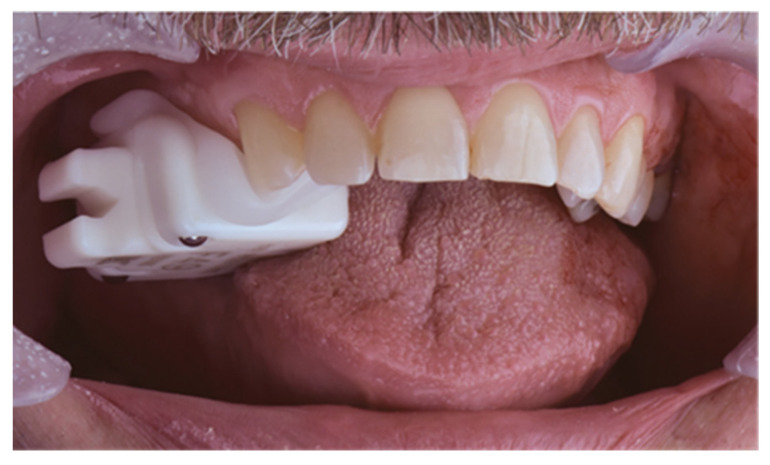
X-clip situated in the patient’s mouth.

Prior to obtaining the preoperative CBCT scan (i-CAT^®^ 3D Imaging System, Imaging Science International Inc, Hatfield, PA, USA), a small registration device, the x-clip (Nobel Biocare™, Zürich, Switzerland), with 3 metal reference points was placed on the teeth contralateral to the implant site. Subsequently, a CBCT scan was made with the x-clip ‘in situ’ (Figure 1) using a voxel size between 0.25 and 0.40 mm and a field of view of 6 cm× 6 cm. All images were exported and saved in DICOM (Digital Imaging and Communications in Medicine) format. To create an intraoral 3D model, an IOS (Trios^®^ 3, 3Shape, Copenhagen, Denmark) was obtained, which was saved as a Standard Tessellation Language (STL) file. As such, additional information regarding soft tissues was acquired.

### 2.3. Virtual Implant Planning

**Figure 2:**
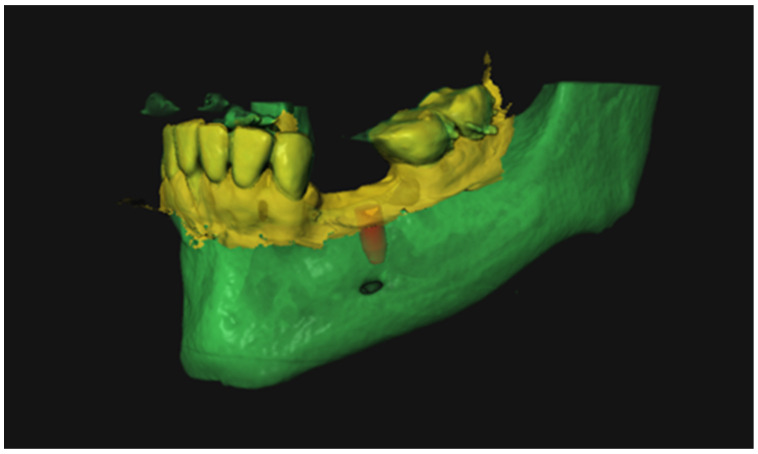
Virtual planning: green represents DICOM data, yellow represents the IOS data, and red represents the planned implant location.

Pre-op DICOM files and the IOS were uploaded in the DTX Studio™ (Nobel BioCare, Zürich, Switzerland) software (Figure 2) and subsequently matched automatically. In this software, the ideal implant location was determined, maintaining a safety margin relative to vital anatomical structures of 1.5 mm. XYZ coordinates for the planned implants tip and shoulder were obtained. Finally, virtual implant planning was imported into the X-guide^®^ system (X-nav, Landsdale, PA, USA).

### 2.4. Implant Placement

**Figure 3:**
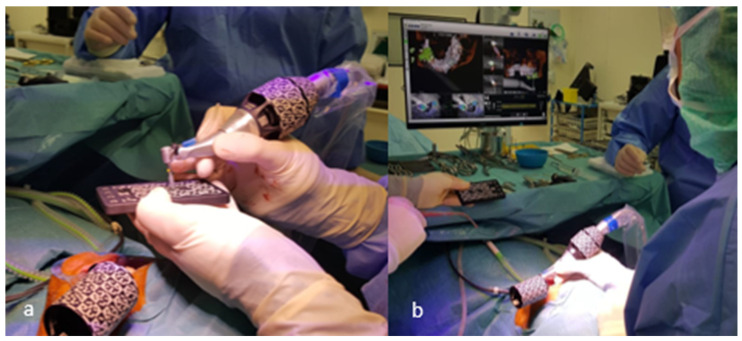
Calibration of the implant drill (**a**) and placement (**b**) of the implant using the X-Guide^®^.

Surgery was performed under local anesthesia using appropriate aseptic and sterile protocols. During the entire procedure, the x-clip was fixed in the exact same location as during CBCT acquisition. Registration, calibration (Figure 3), and system checks were conducted before starting the surgery, as described in the X-Guide^®^ manual. The X-Guide^®^ uses the x-clip as a reference to determine the location of the implant drill as projected during preoperative planning. Osteotomies were prepared at a maximum of 1500 rpm and guided in real time by indicating the desired drilling pathway on the computer screen. An extra calibration process was completed preceding the use of each new drill. Prior to the preparation of the implant placement, no punching of the gingival tissues was performed. NobelParallel^®^ Conical Connection (Nobel Biocare™, Zürich, Switzerland) implants or Nobel Active^®^ (Nobel Biocare™, Zürich, Switzerland) implants were installed. All implants were placed by the same operator (J.L.), who was not involved in data collection and analysis.

### 2.5. Analysis of 3D Imaging Based on Postoperative CBCT and IOS

Immediately after the implant’s placement, a scan abutment (Nobel Biocare, Zürich, Switzerland) was screwed onto the installed implants in order to obtain the postoperative IOS. Subsequently, the scan abutment was replaced with a healing abutment. A postoperative CBCT was obtained using the same settings and parameters as in the preoperative scans. Pre- and postoperative data (pre-op CBCT, post-op CBCT, pre-op IOS, and post-op IOS) were imported in the 3DMedX^®^ software (v1.2.13.2, 3D Lab Radboudumc Nijmegen, The Netherlands) together with the planned implant location.

**Figure 4:**
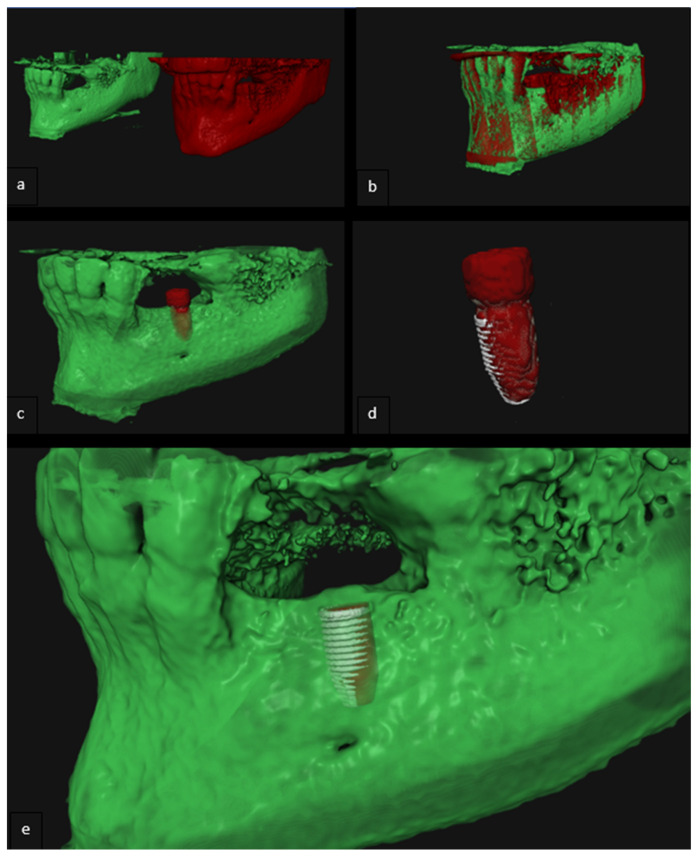
(**a**–**e**) The evaluation of the accuracy of implant placements based on postoperative CBCT. (**a**) 3D model derived from preoperative CBCT scan (green) and postoperative CBCT scan (red); (**b**) Voxel-based matching of the pre- and postoperative 3D model; (**c**) Segmented implant (red) of the postoperative 3D model; (**d**) Voxel-based matching of segmented implant and DICOM model implant (white); (**e**) 3D model of the jaw with the virtually planned implant (red) and the DICOM model corresponding with the placed implant (white).

Pre- and postoperative CBCT images were matched using Voxel-Based Registration (VBR) [8]. Subsequently, a 3D model of the implant was segmented from the registered postoperative CBCT scan. Hereafter, a DICOM model of the implant (with the same diameter and length) was imported into the dataset and roughly aligned with the previously segmented postoperative implant model. After this initial alignment, a VBR procedure was applied to match the DICOM model of the implant accurately with the installed implant (Figure 4a–e).

**Figure 5:**
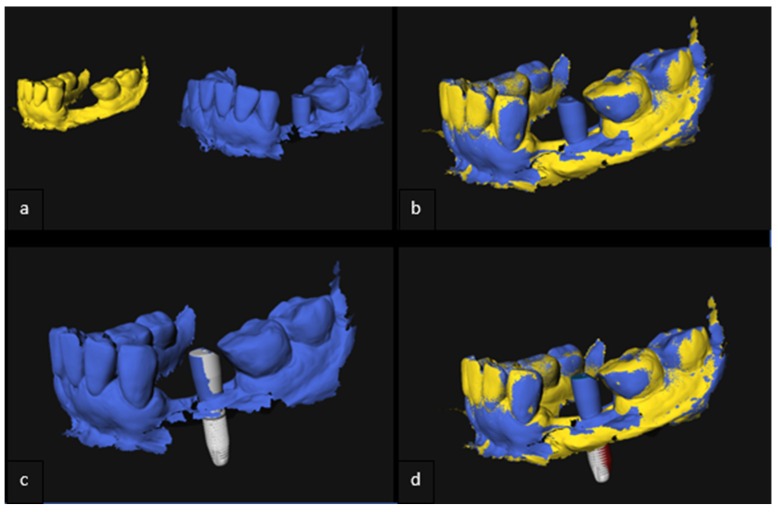
(**a**–**d**) Evaluation of accuracy of implant placement based on postoperative IOS. (**a**) Preoperative (yellow) and postoperative (blue) IOS 3D model; (**b**) Surface based registration of the pre- and postoperative 3D-model; (**c**) Surface based registration of implant model with scan-abutment (white); (**d**) 3D model of the jaw with planned (red) and placed (white) implant.

To compare the postoperative IOS with the preoperative implant planning, first, a Surface-Based Registration (SBR) using the Iterative Closest Point (ICP) algorithm of the preoperative and postoperative IOS took place, based on the patient’s own dentition as reference points excluding scan abutments. To visualize the implant on the postoperative IOS, a computer model, depicting the implant with the scan abutment on top, was loaded into 3DMedX^®^. SBR based on the scan abutment took place to import the implant model in the postoperative IOS. This resulted in a superimposition of the clinically placed implant over the virtually planned implant, as projected on the CBCT scan (Figure 5a–d).

### 2.6. Implant Validation

**Figure 6:**
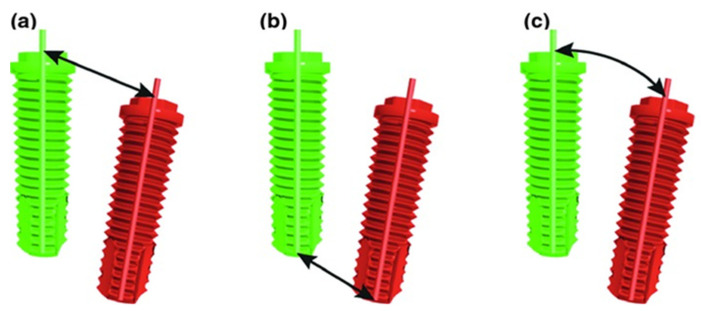
Implant placement deviations in 3D display: (**a**) deviation at the shoulder, (**b**) deviation at the tip, and (**c**) angular deviation.

After analyses and the matching of preoperative data with either the postoperative CBCT or the postoperative IOS, coordinates of the shoulder and tip of the placed implants were determined using 3DMedX^®^ and MATLAB© (R2020b, The MathWorks, Inc., Natick, MA, USA) software. This resulted in two sets of x-, y-, and z-values, one set determined by means of postoperative IOS images and one set by means of postoperative CBCT images. Comparing these values with the coordinates of the planned implant position provided information on three aspects of implant placements, as displayed in Figure 6.

(a) Deviation in implant shoulder in millimeters (mm): three-dimensional distance between shoulder of planned and placed implant, measured from the axis;
(b) Deviation in implant tip in millimeters (mm): three-dimensional distance between tip of planned and placed implant, measured from the axis;
(c) Angular deviation in degrees (°): largest angle between the central, longitudinal axis of planned and realized implant positions.

Additionally, using the Implant Position Orthogonal Projection (IPOP) method, validated by Verhamme et al. [9], deviations were projected along the mesiodistal, buccolingual/palatal planes. To do so, six points were marked on the digital model of the dental arch, resulting in a curve corresponding with the dental arch. By means of both a plane perpendicular and a plane tangent to this arch at the place of the placed implant, information was obtained on the deviation of the implant’s placement, as projected in the mesio–distal (MD) plane and the bucco-lingual/bucco-palatal (BL/BP) plane. This was performed for the data extracted from both the CBCT scan and IOS. By means of the IPOP method, deviations in implant depth on both planes were also determined:

### 2.7. Statistical Analysis

Statistical analysis of the data was performed using SPSS^®^ software (v27, IBM Corp. Armonk, NY, USA). The differences between implant position determined by either CBCT scan or IOS were statistically analyzed using a paired t-test and were found significant if the *p*-value was <0.05:

## 3. Results

In total, 23 implants were placed in 16 patients (11 males and 5 females) with a mean age of 49 years (range 24–78 years). Nine patients received one implant for single-tooth replacement, six patients received two implants, both for single-tooth replacement, and one patient received two implants for an implant-supported bridge. Locations of all individual cases are displayed in Table 1. Deviations between planned and placed implants are displayed in Table 2. Mean deviations are based on absolute values.

**Figure 7:**
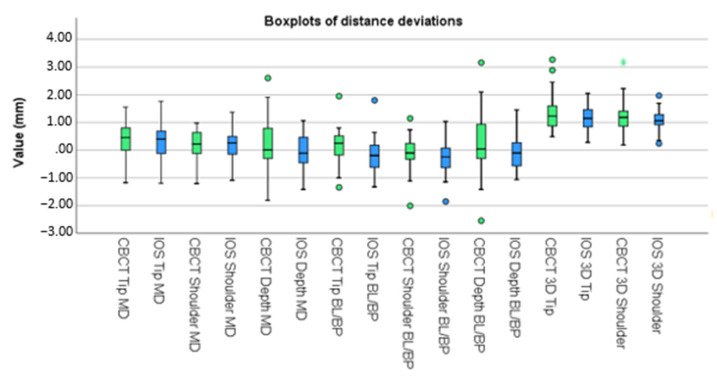
Boxplots of distance deviations.

**Figure 8:**
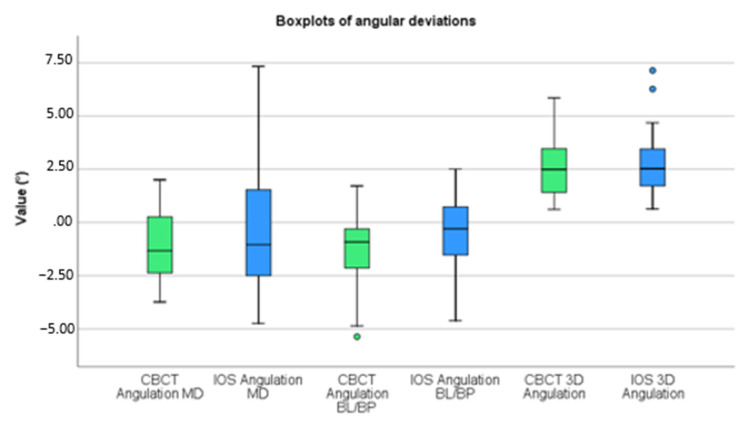
Boxplots of angular deviations.

A paired t-test was performed and discrepancies between accuracy determinations by IOS and by CBCT were analyzed (Table 3). Before calculating discrepancies, mesial, lingual/palatal, and counterclockwise deviations were given a positive value, and distal, buccal, and clockwise deviations were labelled with a negative value. Boxplots of these deviations are displayed in Figure 7 and Figure 8. To test whether the assumption that the small size of each cluster and the correlation between the measurement error between two different implants is weak, we repeated our analysis with multilevel regression analysis that allowed for clustering. This analysis virtually produced identical results.

Tip, shoulder, angular, and depth deviations, as projected on the MD plane and the depth deviation as projected on the BL/BP plane, were all statistically insignificant (*p* > 0.05). Tip, shoulder, and angular deviations as projected on the BL/BP plane were all statistically significant (*p* < 0.05). These deviations displayed a *p*-value of, respectively, 0.000, 0.011, and 0.002.

## 4. Discussion

As computer-guided implant surgery was introduced in oral implantology, clinicians became aware of the relevance of accuracy. Generating a second, postoperative CBCT scan was previously the only possibility to evaluate implant placement accuracies. The introduction of IOS in dentistry led to the suggestion that an IOS could also be used to evaluate implant placements and thus avoids the need of a second postoperative CBCT scan.

CBCT and IOS validation methods displayed a mean absolute deviation, as compared to implant planning, of the implant shoulder in 3D orientation of, respectively, 1.27 and 1.06 mm; the implant tip displayed a deviation of, respectively, 1.37 and 1.19 mm and an angular deviation of, respectively, 2.63 and 2.84 degrees. This falls in line with the other recent literature regarding the implant placement accuracy of dynamic guided implant surgery [10,11,12,13].

To analyze deviations in 3D between IOS and CBCT scans, one could suffice with only calculating the differences between the achieved implant locations between these two imaging types. Since the direction of deviations is also clinically relevant, we focused on the difference between planned and achieved implant positions and, subsequently, defined the direction of the deviations by means of the IPOP method. Mesial, lingual/palatal, and counterclockwise deviations were given a positive value, implicating that distal, buccal, and clockwise deviations were labelled with a negative value. As a result, statistical analysis became feasible.

On the MD plane, no significant differences were found between validation with a postoperative IOS and CBCT scan. On the BL/BP plane, significant differences were found for tip deviations, shoulder deviations, and angular deviations. It concerns only minor differences of, respectively, 0.25 mm, 0.12 mm (both indicating that the IOS implant projection was more to the buccal side), and of −0.81° (the IOS implant projected a more counterclockwise rotation). One must keep in mind that deviations less than 0.25 mm are clinically irrelevant.

**Figure 9:**
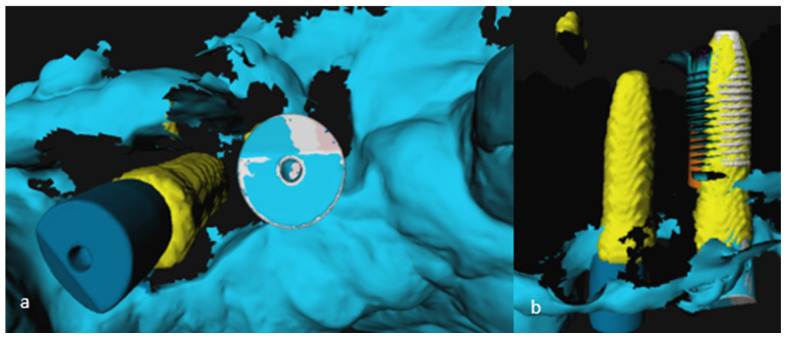
Occlusal (**a**) and subcrestal (**b**) view of two implants.

However, deviations larger than 1 mm are relevant indeed. One case displayed a discrepancy at the tip of 1.69 mm in MD direction and a difference in angulation of 6.26°. In this specific case, the postoperative IOS lacked information about soft tissues surrounding the scan abutment. Although the matching procedure on the scan abutment itself went well, in the end, this missing information led to a miscalculation by the IOS software with respect to the proper location of the scan abutment, as depicted in Figure 9. The matching of the IOS (light blue) and scan abutment DICOM model (grey) shows no errors at the left implant. However, the mesial implant, as segmented from the postoperative CBCT (yellow) and the corresponding DICOM implant model (white), does show a clear deviation between the CBCT model and the IOS model (green blue). This confirms that missing information relative to soft tissues indeed affects optimal matching for determining the implant’s position.

The sample size of this study is, with a total of 23 implants in 16 participants, relatively small.

For CBCT imaging, a voxel size between 0.25 mm and 0.4 mm was used. The voxel size could influence linear measurements; however, the literature has stated that these differences are not found to be statistical significant [14,15].

VBR and SBR procedures were almost entirely automated, with only the initial alignment of the images carried out manually. The studies of Nada et al. [4] and Baan et al. [16] also compared interobserver variabilities in SBR and showed no significant differences, meaning that matching procedures are highly reproducible.

Regarding the accuracy of both matching procedures, the literature states that VBR displays less variability than SBR. However, differences between both methods were found to be non-significant [5,17]. This indicates that determinations in 3D models by means of SBR and in scans by means of VBR can be compared with each other.

Additionally, a visual check of the VBR between segmented implant and implant model showed an accurate match in most cases. However, in two cases, there was a clear deviation visible between the two tips. These tip deviations obviously influence accuracy results. Shoulders and angulations showed no clear deviations in all cases.

Regarding the accuracy of the IOS and, in particular, the Trios^®^ 3, Pattamavilai and Ongthiemsak [18] and Amornvit et al. [19] showed that this scanner produces an accurate and true representation of the real-life intraoral situation.

In an in vitro setting, Zhou et al. [20] claimed that no significant differences were found between implant location determinations by CBCT or IOS. However, this study compared deviations between postoperative imaging, whereas our study compared deviations between preoperative planning and postoperative implant locations. Furthermore, Zhou et al. [20] matched 3D imaging by manually marking fiducial points. Incorporating manual steps into the matching process allows observational errors. In contrast, in our study, matching and all calculations were automatically conducted. Within the IPOP method, the only manual step is to mark six points on the dental arch, which have been proven to have no significant influence on the accuracy of implant placements [9].

Franchina et al. [21] also compared IOS to CBCT for implant placement accuracy determination in an in vitro setting. This study corroborated that IOS is an alternative relative to CBCT to determine implant placement accuracy.

To our knowledge, only the study of Skjerven et al. [22] compared IOS to CBCT as a method for implant placement accuracy in vivo. They also validated IOS as an alternative to CBCT to determine implant placement accuracies. However, they only measured absolute deviations between implant planning and placement. Again, if the deviations are not corrected for the direction of the deviation, including bucco-lingual and mesio-distal directions, the outcome has hardly any clinical significance.

Furthermore, at least two in vivo studies [23,24] already determined implant placement accuracies by postoperative IOS. However, one of these studies by Derksen et al. [23] stated that additional studies to compare both accuracy evaluation methods are necessary to confirm that a postoperative IOS is a valid alternative to a postoperative CBCT for determining implant placement accuracy.

Future developments in software design will introduce fully automated accuracy-determination processes, enabling the surgeon to determine the accuracy of implant placement preoperatively.

Future studies could focus on using IOS as a method to evaluate implant placement accuracy in fully edentulous patients. Since the IOS does not have any teeth as reference points in these cases, one would think that IOS is not suited for fully edentulous patients.

Additionally, a study with the same design as this study could be carried out again but on a larger group of patients. A power analysis before conducting the study could indicate how many patients are needed and adds more power to the study’s findings.

Besides using IOS to assess implant placement accuracies, other non-invasive methods could be further researched in a controlled clinical trial and be compared to CBCT and IOS to determine the accuracy of each of these methods.

## 5. Conclusions

Our results show that a postoperative IOS is a validated alternative to a postoperative CBCT scan for determining implant placement accuracy.

There were no significant deviations found between CBCT and IOS on the MD-plane and only relatively small, significant deviations on the BL/BP-plane. However, since there are but a few clinical studies comparing IOS to CBCT for the evaluation of implant placement accuracy, additional research is needed to support our statements.

## Figures and Tables

**Table 1:** Implant location of all cases.

Case	Mandible/Maxilla	Implant Location
1	Maxilla	11
2	Mandible	36
3	Maxilla	15
4	Maxilla	16
5.1	Maxilla	13
5.2	Maxilla	12
6	Maxilla	21
7.1	Maxilla	12
7.2	Maxilla	14
8.1	Mandible	46
8.2	Mandible	47
9	Maxilla	12
10	Mandible	35
11.1	Maxilla	13
11.2	Maxilla	23
12.1	Mandible	36
12.2	Mandible	37
13	Maxilla	13
14.1	Maxilla	11
14.2	Maxilla	21
15.1	Maxilla	24
15.2	Maxilla	25
16	Maxilla	21

**Table 2:** Mean difference between planned and placed implants, as determined by CBCT and IOS.

		Mean (CBCT)	Standard Deviation (CBCT)	Mean (IOS)	Standard Deviation (CBCT)
Mesio-Distal plane	Tip (mm)	0.601	0.460	0.685	0.466
	Shoulder (mm)	0.473	0.350	0.486	0.348
	Angular (°)	1.643	1.220	2.288	1.608
	Depth (mm)	0.151	1.016	−0.045	0.692
Bucco-Lingual/Bucco-palatal plane	Tip (mm)	0.535	0.455	0.552	0.454
	Shoulder (mm)	0.500	0.489	0.549	0.451
	Angular (°)	1.755	1.555	1.421	1.169
	Depth (mm)	0.209	1.206	−0.045	0.680
3D plane	Tip (mm)	1.369	0.746	1.186	0.484
	Shoulder (mm)	1.265	0.773	1.057	0.429
	Angular (°)	2.625	1.494	2.835	1.595

Cone-Beam Computed Tomography (CBCT) and intraoral scan (IOS).

**Table 3:** Statistical analysis of net deviations between CBCT and IOS.

		Mean	Standard Deviation	95% Confidence Interval of the Difference		*p*-Value
				Lower	Upper	
Mesio-Distal Plane	Tip (mm)	0.09	0.54	−0.14	0.33	0.419
	Shoulder (mm)	0.01	0.35	−0.14	0.16	0.910
	Angular (°)	−0.55	2.34	−1.56	0.46	0.273
	Depth	0.20	0.85	−0.17	0.57	0.280
Bucco-Lingual/Bucco-palatal plane	Tip (mm)	0.25	0.23	0.15	0.34	0.000 *
	Shoulder (mm)	0.12	0.20	0.03	0.21	0.011 *
	Angular (°)	−0.81	1.10	−1.28	−0.33	0.002 *
	Depth	0.17	0.88	−0.21	0.55	0.372

* Statistical significance.

## Data Availability

The data presented in this study will be made openly available in DANS EASY data repository after the publication of this manuscript.
